# Diagnosis and management of progressive ataxia in adults

**DOI:** 10.1136/practneurol-2018-002096

**Published:** 2019-05-02

**Authors:** Rajith Nilantha de Silva, Julie Vallortigara, Julie Greenfield, Barry Hunt, Paola Giunti, Marios Hadjivassiliou

**Affiliations:** 1 Department of Neurology, Essex Centre for Neurological Sciences, Queen’s Hospital, Romford, UK; 2 Ataxia UK, London, UK; 3 Department of Clinical and Movement Neurosciences, Ataxia Centre, UCL Institute of Neurology, London, UK; 4 Academic Department of Neurosciences, Sheffield Teaching Hospitals NHS Trust and University of Sheffield, Sheffield, UK

**Keywords:** progressive ataxia, cerebellar disease, diagnosis and management, molecular genetics, immunity

## Abstract

Progressive ataxia in adults can be difficult to diagnose, owing to its heterogeneity and the rarity of individual causes. Many patients remain undiagnosed (‘idiopathic’ ataxia). This paper provides suggested diagnostic pathways for the general neurologist, based on Ataxia UK’s guidelines for professionals. MR brain scanning can provide diagnostic clues, as well as identify ‘structural’ causes such as tumours and multiple sclerosis. Advances in molecular genetics, including the wider and cheaper availability of ‘next-generation sequencing’, have enabled clinicians to identify many more cases with a genetic cause. Finally, autoimmunity is probably an under-recognised cause of progressive ataxia: as well as patients with antigliadin antibodies there are smaller numbers with various antibodies, including some associated with cancer. There are a few treatable ataxias, but also symptomatic treatments to help people with the spectrum of complications that might accompany progressive ataxias. Multidisciplinary team involvement and allied health professionals’ input are critical to excellent patient care, including in the palliative phase. We can no longer justify a nihilistic approach to the management of ataxia.

## Introduction

Ataxia, or lack of coordination, is a common manifestation of various neurological conditions, including stroke, brain tumour, multiple sclerosis, traumatic brain injury, toxicity, infection (including following varicella) and congenital cerebellar defects. Its evolution can be acute, subacute, episodic or chronic. Progressive ataxias frequently cause diagnostic uncertainty in general neurological practice, and many cases remain undiagnosed (or ‘idiopathic’). This review aims to provide general neurologists with helpful pathways for diagnosing and managing adults with progressive ataxias. Recent advances in molecular genetics have enabled the diagnosis of many more cases with a genetic cause. Autoimmunity may also be important (but under-recognised) in causing some progressive cerebellar ataxias. There are no current treatments to halt the progression of most forms of chronic ataxia (with a few notable exceptions) but there have been recent advances in disease-modifying interventions. Ataxia management warrants a broad and multidisciplinary approach.

In this review, we explore in detail the progressive ataxias in adults, providing guidance on how to diagnose and to manage patients (and their families). Our advice is based on Ataxia UK’s guidelines on the management of the ataxias (third edition July 2016), to which we have contributed (https://www.ataxia.org.uk/Handlers/Download.ashx?IDMF=261e0aa4-5ca0-4b90-9db0-1ecb6ef8738a).^1^ Our emphasis is clinical, guiding the busy general neurologist to a precise diagnosis, identifying pitfalls to avoid and outlining best practice in managing patients.

### Types of ataxia

The ataxias may be broadly divided into those that are genetic (with or without a family history) and those that are acquired/degenerative. ‘Sporadic’ ataxia implies there is no family history. Acquired progressive ataxias can be immune mediated (eg, paraneoplastic spinocerebellar degeneration, gluten ataxia), degenerative (eg, cerebellar variant of multiple systems atrophy (type C)), caused by deficiency states (eg, vitamin B_12_, vitamin E, and so on), toxicity (eg, alcohol-related ataxia, phenytoin), or associated with infections (HIV, sporadic Creutzfeldt-Jakob disease, progressive multifocal leucoencephalopathy, and so on). Inherited ataxias can have autosomal dominant, autosomal recessive, X-linked or mitochondrial (maternal) inheritance. Metabolic disorders (eg, Niemann-Pick type C, Tay-Sachs disease), even though ‘inherited’, can present as late-onset ataxia with no family history, emphasising the need for careful clinical scrutiny and comprehensive and appropriate laboratory testing.

### Clinical presentation

Patients with ataxia report clumsiness, unsteadiness, incoordination and slurred speech. Rarely, they experience oscillopsia. When examined, there may be one or more of the following signs^2^:

- Gait ataxia and impaired sitting balance (usually late in the disease).
- Gaze-evoked nystagmus, jerky (saccadic) pursuit and hypo/hypermetropic saccades.
- Dysarthria.
- Intention tremor.
- Dysmetria.
- Dysdiadochokinesis.

The signs rarely indicate the cause of the patient’s ataxia but occasionally there are very helpful hints.

- ** *Reflexes* are usually reduced or absent in Friedreich’s ataxia, ataxia associated with vitamin E deficiency, ataxia with oculomotor apraxia type 2 and spinocerebellar ataxia (SCA) type 2. Reflexes are present, even brisk, in patients with most of the dominant SCAs and patients with multiple systems atrophy type C.
- *Eye movements*. Oculomotor apraxia can develop in ataxia-telangiectasia and in ataxia with oculomotor apraxia type 1 (although rare in this condition) and type 2. Slow saccades are typical of SCA type 2.
- *Postural hypotension* (often with impotence and urinary urgency/incontinence) points towards multiple systems atrophy type C.
- *Tendon xanthomas* (and early-onset cataracts) suggest cerebrotendinous xanthomatosis.
- *Abnormal visually enhanced vestibulo-ocular reflexes* (and pathological head impulse test responses) are characteristic of cerebellar ataxia with neuropathy and vestibular areflexia syndrome (CANVAS), which also has a sensory neuropathy/neuronopathy.^39^

The age of onset and the rate of ataxia progression are perhaps the two most useful clinical features pointing to the cause.

- *Age of onset.* Ataxia may appear first in infancy (reflecting a congenital or developmental cause, often genetic) or develop before aged 20 years (early-onset ataxia). Most early-onset ataxias prove to be genetic (usually autosomal recessive or mitochondrial inheritance), even if there is no family history. Dominant ataxias (including the SCAs) tend to present later (from the third and fourth decades onwards). However, this rule does not always hold: Friedreich’s ataxia can have a late onset (with intact or even brisk reflexes) and also some SCAs have very young-onset forms (associated with large CAG repeat expansions). CANVAS, despite recessive inheritance, presents unusually late (in middle age).^39^
- *Rapid progression* (within weeks to months) is characteristic of paraneoplastic spinocerebellar degeneration and sporadic Creutzfeldt-Jakob disease. Multiple systems atrophy type C can also advance faster than other progressive neurodegenerative ataxias (including inherited types), which generally progress over many years.

There are validated measures (such as the ‘scale for the assessment and rating of ataxia’, table 1) to monitor the rate of progression serially.^3^ Therapeutic trials require researchers to use rating scales, which have as their endpoint the improvement or the halting of progression of ataxia.

**Table 1:** Scale for the assessment and rating of ataxia (SARA)

**1. Gait**			**2. Stance**		
Proband is asked (1) to walk at a safe distance parallel to a wall including a half-turn (turn around to face the opposite direction of gait) and (2) to walk in tandem (heels to toes) without support. 0—Normal, no difficulties in walking, turning and walking tandem (up to one misstep allowed) 1—Slight difficulties, only visible when walking 10 consecutive steps in tandem 2—Clearly abnormal, tandem walking >10 steps not possible 3—Considerable staggering, difficulties in half-turn, but without support 4—Marked staggering, intermittent support of the wall required 5—Severe staggering, permanent support of one stick or light support by one arm required 6—Walking >10 m only with strong support (two special sticks or stroller or accompanying person) 7—Walking <10 m only with strong support (two special sticks or stroller or accompanying person) 8—Unable to walk, even supported			Proband is asked to stand (1) in natural position, (2) with feet together in parallel (big toes touching each other) and (3) in tandem (both feet on one line, no space between heel and toe). Proband does not wear shoes, eyes are open. For each condition, three trials are allowed. The best trial is rated. 0—Normal, able to stand in tandem for >10 s 1—Able to stand with feet together without sway, but not in tandem for >10 s 2—Able to stand with feet together for >10 s, but only with sway 3—Able to stand for >10 s without support in natural position, but not with feet together 4—Able to stand for >10 s in natural position only with intermittent support 5—Able to stand >10 s in natural position only with constant support of one arm 6—Unable to stand for >10 s even with constant support of one arm		
Score			Score		
**3. Sitting**			**4. Speech disturbance**		
Proband is asked to sit on an examination bed without support of feet, eyes open and arms outstretched to the front. 0—Normal, no difficulties sitting >10 s 1—Slight difficulties, intermittent sway 2—Constant sway, but able to sit >10 s without support 3—Able to sit for >10 s only with intermittent support 4—Unable to sit for >10 s without continuous support			Speech is assessed during normal conversation. 0—Normal 1—Suggestion of speech disturbance 2—Impaired speech, but easy to understand 3—Occasional words difficult to understand 4—Many words difficult to understand 5—Only single words understandable 6—Speech unintelligible/anarthria		
Score			Score		
**5. Finger chase**			**6. Nose–finger test**		
*Rated separately for each side* Proband sits comfortably, if necessary with their feet and trunk supported. Examiner sits in front of proband and performs five consecutive sudden and fast-pointing movements in unpredictable directions in a frontal plane, at about half of proband’s reach. Movements have an amplitude of 30 cm and a frequency of one movement every 2 s. Proband is asked to follow the movements with his index finger, as fast and precisely as possible. Average performance of last three movements is rated. 0—No dysmetria 1—Dysmetria, under/overshooting target <5 cm 2—Dysmetria, under/overshooting target <15 cm 3—Dysmetria, under/overshooting target >15 cm 4—Unable to perform 5 pointing movements			*Rated separately for each side* Proband sits comfortably, if necessary with their feet and trunk supported. Proband is asked to point repeatedly with the index finger from his nose to examiner’s finger, which is in front of the proband at about 90% of proband’s reach. Movements are performed at moderate speed. The average performance of movements is rated according to the amplitude of the kinetic tremor. 0—No tremor 1—Tremor with an amplitude <2 cm 2—Tremor with an amplitude <5 cm 3—Tremor with an amplitude >5 cm 4—Unable to perform 5—Pointing movements		
Score	Right	Left	Score	Right	Left
Mean of both sides (R+L)/2			Mean of both sides (R+L)/2		
**7. Fast alternating hand movements**			**8. Heel–shin slide**		
*Rated separately for each side* Proband sits comfortably, if necessary, with the feet and trunk supported. The proband is asked to perform 10 cycles of repetitive alternation of pronations and supinations of the hand on the thigh as fast and as precisely as possible. The examiner demonstrates the movement at about 10 cycles within 7 s. Exact times for movement execution have to be taken. 0—Normal, no irregularities (performs <10 s) 1—Slightly irregular (performs <10 s) 2—Clearly irregular, single movements difficult to distinguish or relevant interruptions, but performs <10 s 3—Very irregular, single movements difficult to distinguish or relevant interruptions, performs >10 s 4—Unable to complete 10 cycles			*Rated separately for each side* Proband lies on examination bed, unable to see his/her legs. Proband is asked to lift one leg, point with the heel to the opposite knee, slide down along the shin to the ankle, and lay the leg back on the examination bed. The task is performed three times. Slide-down movements should be performed within 1 s. If proband slides down without contact to shin in all three trials, rate 4. 0—Normal 1—Slightly abnormal, contact to shin maintained 2—Clearly abnormal, goes off shin up to three times during three cycles 3—Severely abnormal, goes off shin four or more times during three cycles 4—Unable to perform the task		
Score	Right	Left	Score	Right	Left
Mean of both sides (R+L)/2			Mean of both sides (R+L)/2		

### Investigation

Brain imaging with MRI (or CT, if MRI is contraindicated) is essential in almost everyone with ataxia. While it is rarely diagnostic, it often provides helpful diagnostic clues, as well as ruling out structural pathology (table 2). The MR scan usually shows cerebellar atrophy, in line with the clinical appraisal.

**Table 2:** Diagnostic clues from MR scanning of the brain

Condition	MR brain scan finding(s)	MRI sequence
*‘Diagnostic’*		
Multiple systems atrophy type C	‘Hot-cross bun’ sign*; pontine atrophy (figure 1)	T2/FLAIR
Fragile X tremor-ataxia syndrome	Middle cerebellar peduncle sign† (figure 2)	T2/FLAIR
Superficial siderosis	Deposition of haemosiderin; cerebellar atrophy (figure 3)	GRE/T2*
Sporadic Creutzfeldt-Jakob disease	High basal ganglia signal; cortical high (and persistent DWI) signal	T2/FLAIR, DWI/ADC
Autosomal recessive spastic ataxia of Charlevoix-Saguenay	Hypointense pontine stripes (figure 4); atrophy of superior cerebellar vermis; thinning of posterior mid-body of corpus callosum	T2/FLAIR
SPG7	High dentate nuclei signal (figure 5)^38^	T2/FLAIR
*‘Suggestive’* (*in familial forms*)		
Friedreich’s ataxia, vitamin E deficiency	Upper cervical cord atrophy; cerebellar atrophy late (figure 6)	T1/T2
Ataxia-telangiectasia, ataxia with oculomotor apraxia, types 1 and 2	Cerebellar atrophy	T1/T2
Autosomal dominant spinocerebellar ataxia	Cerebellar and pontine atrophy	T1/T2

*Also occurs in autosomal dominant spinocerebellar ataxia.

†Can occur in multiple systems atrophy type C.

ADC, apparent diffusion coefficient; DWI, diffusion-weighted imaging; FLAIR, fluid-attenuated inversion recovery; GRE, gradient echo; SPG7, spastic paraplegia 7.

**Figure 1:**
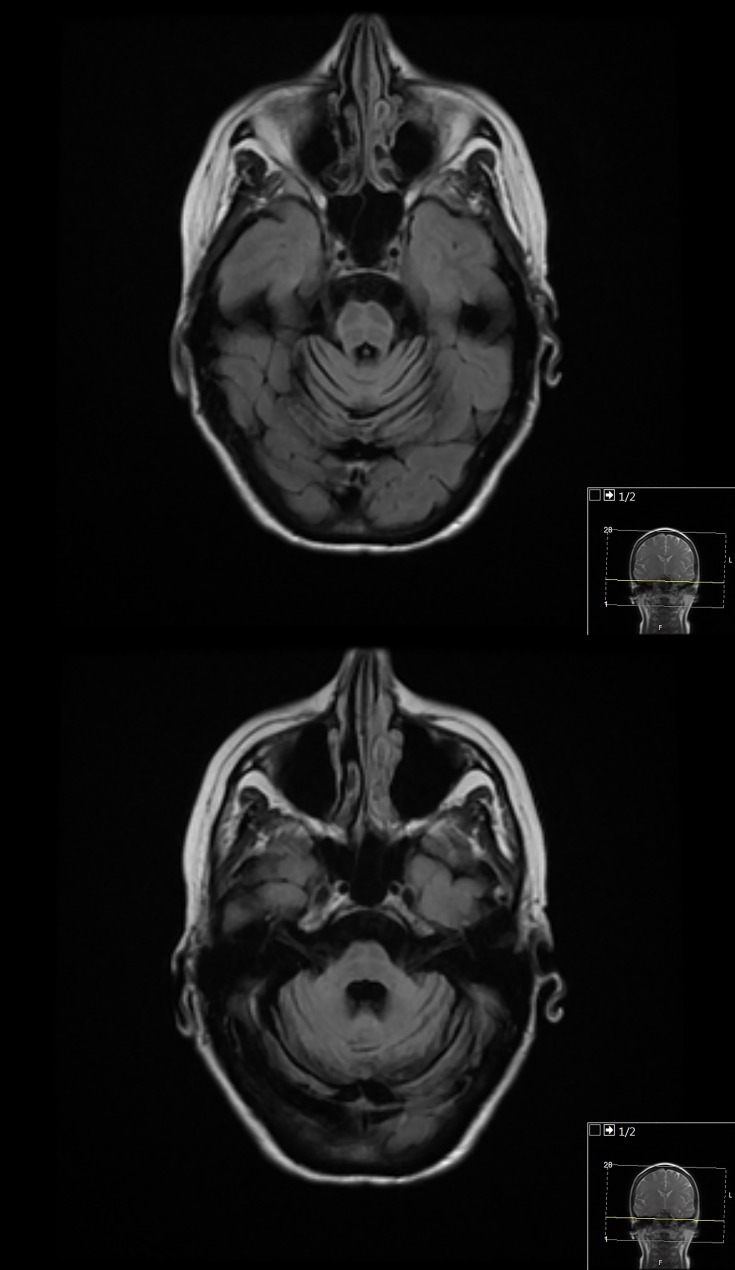
Fluid-attenuated inversion recovery (FLAIR) axial image of the brain, showing pontine and cerebellar atrophy, and ‘hot-cross bun’ sign, in a patient with multiple systems atrophy type C (MSA-C). Note the narrow middle cerebellar peduncles.

**Figure 2:**
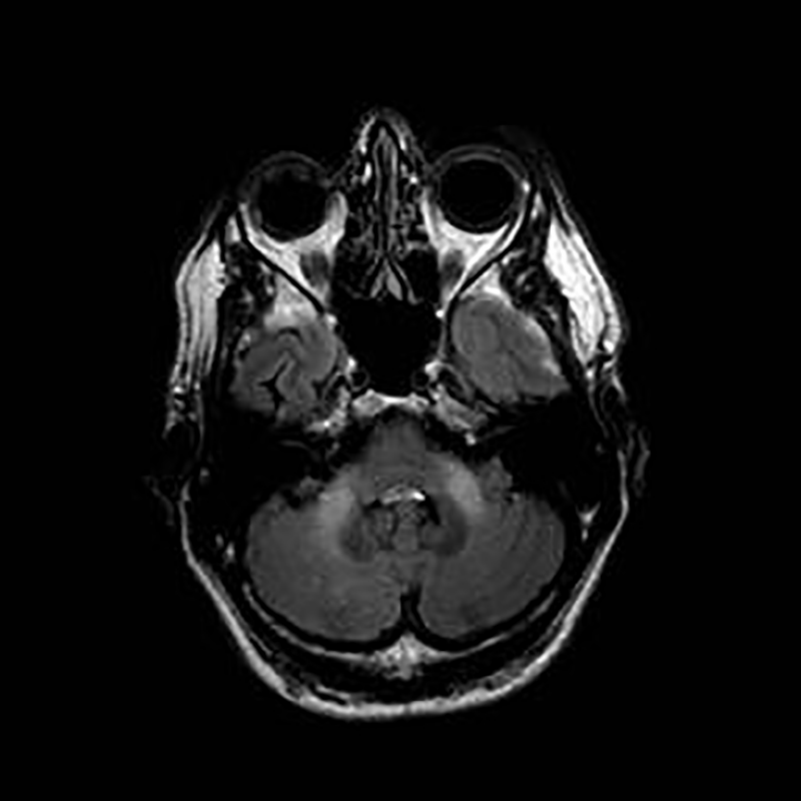
Axial T2-fluid-attenuated inversion recovery (FLAIR) image of the brain, showing high signal in the middle cerebellar peduncles, in a case of fragile X tremor-ataxia syndrome (FXTAS).

**Figure 3:**
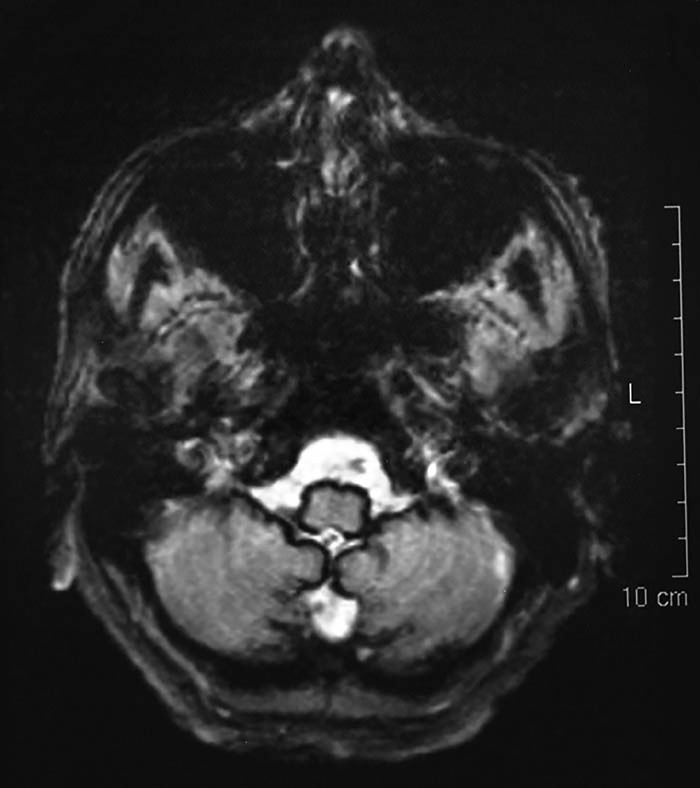
Axial T2-weighted MRI of the brain, showing haemosiderin deposition around the medulla and cerebellum, in a case of superficial siderosis. (Gradient echo (GRE)/T2* imaging would have demonstrated these changes more vividly.)

**Figure 4:**
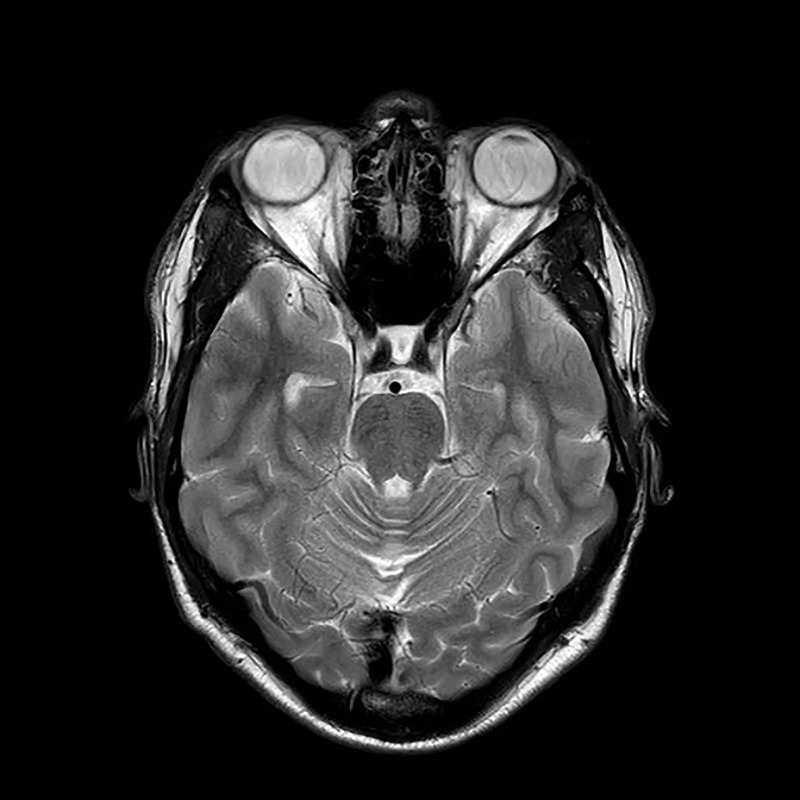
Axial T2-weighted MRI of the brain in a case of autosomal recessive spastic ataxia of Charlevoix-Saguenay (ARSACS) showing pontine hypointense (‘tigroid’) stripes.

**Figure 5:**
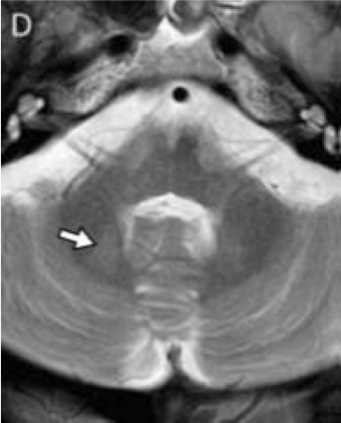
Axial T2-weighted MRI of the brain in a case of hereditary spastic paraplegia 7 (SPG7) showing hyperintensity of the dentate nuclei (arrowed).

**Figure 6:**
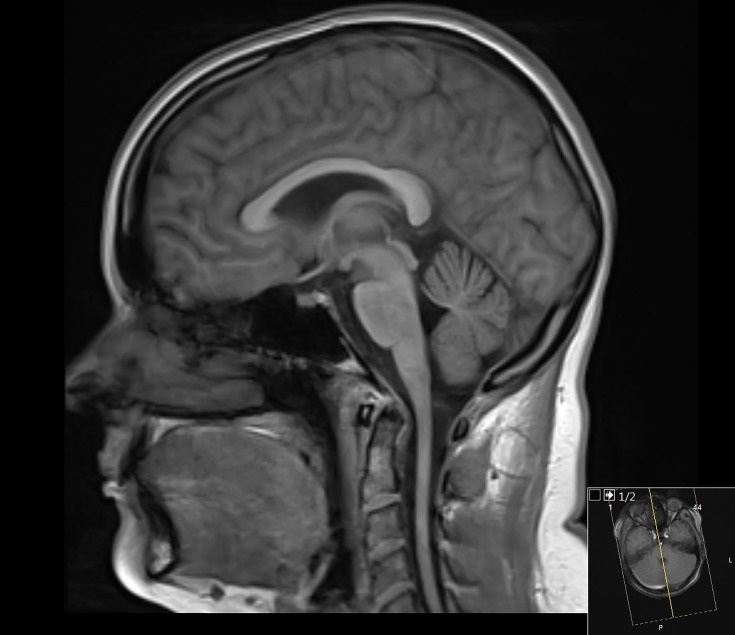
Sagittal T1-weighted MRI of the brain and upper cervical cord in a 26-year-old woman with ataxia. There is relative preservation of the brainstem and cerebellum, but thinning of upper cervical cord. (Friedreich’s ataxia (FRDA) was confirmed in her genetically.)

### Diagnostic tests

Table 3 shows the investigations useful for patients presenting with ataxia, listed as first, second and third lines, based on the guidelines.

**Table 3:** Diagnostic investigations in adults

Primary care	Serum urea and electrolytes, serum creatinine, full blood count ESR/C-reactive protein	Liver enzymes, serum γ-GT, thyroid function tests Vitamin B_12_	Serum folate, plasma glucose, chest X-ray
Secondary care (first line)	αFP Blood film Caeruloplasmin/copper Coeliac disease screen, serum creatine kinase Genetic tests for Friedreich’s ataxia, SCA 1, 2, 3, 6, 7 (12, 17) and fragile X tremor-ataxia syndrome	Lactate Lipid-adjusted vitamin E and lipoproteins Lumbar puncture (cells, protein, glucose, cytology, oligoclonal bands, lactate, ferritin) MR scan of brain and cervical spine	Anti-Hu/Yo and other paraneoplastic antibodies Anti-GAD antibody Anti-voltage-gated calcium channel antibody CT scan of chest, abdomen, pelvis 14-3-3 and other proteins in CSF (prion diseases)
Secondary care (second line)	Cholestanol Plasma oxysterols Bile acids Coenzyme Q10 (ubiquinone) Electroencephalography Very-long-chain fatty acids	Muscle biopsy Ophthalmology/optical coherence tomography Peripheral nerve conduction studies Phytanic acid	Remaining genetic tests (next-generation sequencing) Total body PET scan White cell enzymes

CSF, cerebrospinal fluid; ESR, erythrocyte sedimentation rate; αFP, alpha-fetoprotein; GAD, glutamic acid decarboxylase; γ-GT, gamma-glutamyltransferase; PET, positron emission tomography; SCA, spinocerebellar ataxia.

First-line studies, such as checking thyroid function, serum B_12_ and folate (and homocysteine) and coeliac serology, could be undertaken in primary care. Most people with ataxia require evaluation in secondary care, preferably by a neurologist. At this point, in addition to cranial (with/without spinal) imaging, patients may need (depending on clinical context) lumbar puncture, electrophysiological testing, CT scan of thorax, abdomen and pelvis, and muscle biopsy. Genetic testing (even if there is no family history) may also be considered at this stage, given how commonly genetic factors cause progressive ataxia. Rapid progression of ataxia (within months) should prompt a search for underlying malignancy, including with serological testing for paraneoplastic antibodies.^4^ A fluorodeoxyglucose positron emission tomography study may be indicated, even if the CT scan of thorax, abdomen and pelvis is normal. The responsible malignancy may be occult and easily missed in the initial imaging. Its identification may enable earlier diagnosis of the cancer, and possibly cure. Some forms of Creutzfeldt-Jakob disease (including inherited forms, such as Gerstmann-Straussler syndrome) can present with progressive ataxia, when again the tempo of deterioration may be relatively rapid. Neurologists frequently infer that alcohol toxicity is causing a patient’s progressive cerebellar ataxia but such cases usually justify further investigation, which often identifies alternative/additional explanations for their ataxia.

### Genetic testing

Genetic diagnoses among patients with progressive inherited ataxia are still evolving, especially with recent rapid advances in molecular genetics. The advent and impact of next-generation sequencing is such that targeted searches for known gene mutations (other than in family members, where this is already fully characterised) may soon become obsolete. Candidate gene testing is still available, for example, in the UK via the UK Genetics Testing Network (https://ukgtn.nhs.uk). Next-generation sequencing panels allow the search for a wider array of ‘ataxia genes’, available from specialist genetic laboratories (Oxford and Sheffield, in the UK). When the diagnosis remains elusive, clinicians should consider exome or whole-genome sequencing.

When undertaking genetics tests, even for diagnostic purposes, clinicians should counsel people with ataxia about the potential implications of a positive result for other family members. Genetic counselling services should be made available for asymptomatic relatives who wish to be tested, after identifying a pathological genetic variant in a patient with progressive ataxia. There may also be important implications on the reproductive choices that patients and at-risk relatives make, based on this knowledge, including in relation to prenatal testing and preimplantation genetic diagnosis.^5^ These services are delivered by clinical genetics services, in liaison with specialist obstetrics/prenatal units.

Next-generation sequencing is already showing great promise for investigating patients with ataxia. There are already ‘ataxia panels’ using parallel sequencing technology but they are limited because they seek only known (ataxia) genes.^6^ Exome sequencing and whole-genome sequencing (the approach adopted by the 100,000 Genomes Project) are increasingly used in clinical practice, and will probably make major contributions to our knowledge of the underlying genetic factors and mechanisms causing ataxia, as well as expanding our diagnostic acumen. A critical issue with these ‘novel’ techniques is the generation of data in the form of variants of unknown significance, and the interpretation of ‘results’ needs significant bioinformatics input. Technologically, too, next-generation sequencing is still not sufficiently reliable to identify large-scale genomic rearrangements, including nucleotide expansions with certainty (or with precision regarding the number of repeats, in the case of expansions). Thus, at present, we need a combination of conventional and novel techniques to investigate familial ataxia.

#### Dominant ataxias

**Figure 7:**
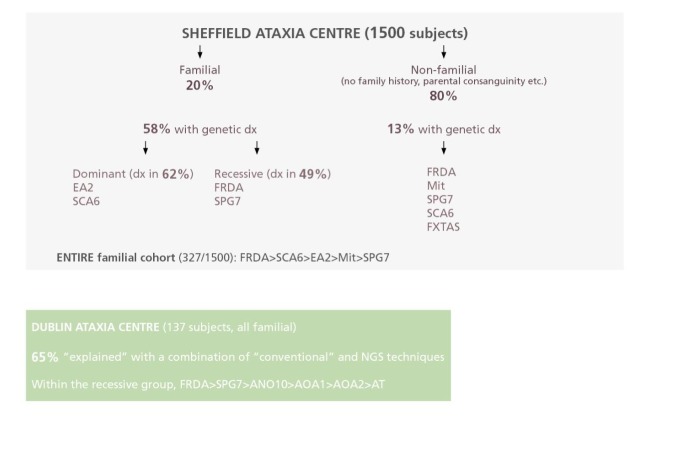
How often is a genetic cause of progressive ataxia identified? Dx, diagnosis; ANO10, ANO10-associated ataxia; AOA1/AOA2, ataxia with oculomotor apraxia types 1 and 2; AT, ataxia-telangiectasia; EA2, episodic ataxia type 2; FRDA, Friedreich’s ataxia; FXTAS, fragile X tremor-ataxia syndrome; Mit, mitochondrial cytopathy; SCA6, spinocerebellar ataxia type 6; SPG7, hereditary spastic paraplegia 7.

There are over 40 dominant currently identified SCAs, some of which may be limited to a small number of families or geographical areas.^7^ SCAs 1, 2 and 3 can be ‘complicated’ by the co-occurrence of cognitive decline, slow saccades, ophthalmoplegia, pyramidal/extrapyramidal signs and neuropathy. Patients with SCA7 usually also have macular degeneration and blindness. Some forms—including SCA7 and dentato-rubro-pallido-luysian atrophy—have extreme repeat expansion instability, and hence a tendency for severe anticipation (particularly when paternally inherited). SCA6 (a relatively ‘pure’ form of SCA) typically has stable CAG repeat expansions during meiosis. The causative gene of SCA6 is also implicated in another form of dominant ataxia, episodic ataxia type 2, and a form of familial hemiplegic migraine (both of which, like SCA6, manifest with progressive ataxia, usually after 50 years). SCA6/episodic ataxia type 2 is the most common dominant ataxia in the British Isles (see figure 7). The genetic mutations underpinning the SCAs are diverse, and include trinucleotide repeat expansions (eg, SCAs 1–3 and 6), non-trinucleotide repeat expansions (eg, SCA10, where there is an intronic pentanucleotide repeat expansion of *ATXN10*), deletions/insertions and missense/nonsense mutations.

#### Recessive ataxias

Friedreich’s ataxia is the most common inherited ataxia in Caucasian populations, with a prevalence of around 1 per 20 000–50 000. Spastic paraplegia 7 (SPG7), a classical cause of hereditary spastic paraparesis, is the next most common recessive ataxia in the UK.^8^ Patients with SPG7 may have only minimal spasticity. Spastic ataxia is also a feature of autosomal recessive spastic ataxia of Charlevoix-Saguenay. In terms of frequency, Friedreich’s ataxia and SPG7 are followed by ataxia with oculomotor apraxia type 2 and ataxia-telangiectasia (which presents less commonly in adulthood). MRI may provide useful clues on the genetic cause (table 2). Clues to ataxia-telangiectasia (and ataxia with oculomotor apraxias) include a high serum alpha-fetoprotein and creatine kinase, and a low serum albumin. Optical coherence tomography can provide support for a diagnosis of autosomal recessive spastic ataxia of Charlevoix-Saguenay.

Despite the growing emphasis on next-generation sequencing in helping to diagnose inherited ataxia,^6^ accurate clinical phenotyping (incorporating relevant laboratory data) remains important. This is especially so when there are ‘variants of uncertain significance’: knowledge and experience must then supplement the bioinformatics pipeline. ‘Deep phenotyping’ is an emerging field where promising biomarkers are used to target genetic testing (which is still expensive). For example, retinal fibre layer thickening (identified using optical coherence tomography) appears to be a sensitive and specific indicator of autosomal recessive spastic ataxia of Charlevoix-Saguenay.^9^ Peripheral electrophysiology may also help define the cause of (recessive) ataxias. There are four categories (after eliminating Friedreich’s ataxia) based on:

1. The absence of neuropathy (mutations in *SYNE1*, *ANO10* and *ADCK3*).
2. The presence of a pure sensory neuronopathy (mutations in *POLG1,* *AVED* and *RFC1*).
3. The presence of an axonal sensorimotor neuropathy (ataxia with oculomotor apraxia types 1 and 2, and cerebrotendinous xanthomatosis).
4. The presence of a demyelinating neuropathy (autosomal recessive spastic ataxia of Charlevoix-Saguenay).^10^

### Relative frequency of genetic forms of ataxia

Figure 7 shows data from two specialist ataxia centres in Sheffield and Dublin, giving an insight to the relative frequencies of the different inherited ataxias in the British Isles. The data also suggest the likelihood of making a specific diagnosis in the specialist setting.^11 12^ However, these proportions vary with geography, reflecting founder gene mutations on different populations.

#### Immunological studies, including testing, for coeliac disease

Autoimmunity is increasingly recognised as a cause of progressive cerebellar ataxia. A cohort of patients from Sheffield with ataxia and positive antigliadin antibodies (a serological marker of gluten sensitivity and coeliac disease) is perhaps the best studied.^13^ In this series, 25% of all sporadic cases were diagnosed with gluten ataxia, although in other centres the proportion recognised is considerably lower.^11^ Given Sheffield’s interest and expertise, testing included serum antigliadin IgG and IgA, antiendomysium and antitransglutaminase antibodies (including TG6 antibodies) and duodenal biopsy. Only half of patients with gluten ataxia have small bowel enteropathy (coeliac disease) and so antigliadin antibodies are essential for diagnosing the remainder. Antibodies against transglutaminase type 6 (TG6) are a potentially useful biomarker of gluten ataxia, and such testing may become commercially viable shortly (zedira.com). Mutations in the transglutaminase 6 gene (*TGM6*) meanwhile are associated with a dominant SCA (SCA35), further supporting a role for TG6 in cerebellar functioning. There is also a well-characterised form of progressive myoclonic ataxia (of Ramsay Hunt) in patients with coeliac disease; most have refractory coeliac disease and need aggressive immunosuppression and chemotherapy.

Other immune-mediated ataxias include paraneoplastic spinocerebellar degeneration, anti-glutamic acid decarboxylase-associated ataxia, and ataxia associated with several other antibodies (anti-voltage-gated calcium channel, dipeptidyl-peptidase-like protein 6 (DPPX), and so on). These frequently present acutely or subacutely. Taken together, these observations raise the possibility that immunity plays a critical role in causing cerebellar injury in some patients with ataxia. Thus, immune modulation may have a possible role in stabilising their disease and preventing progression.

**Figure 8:**
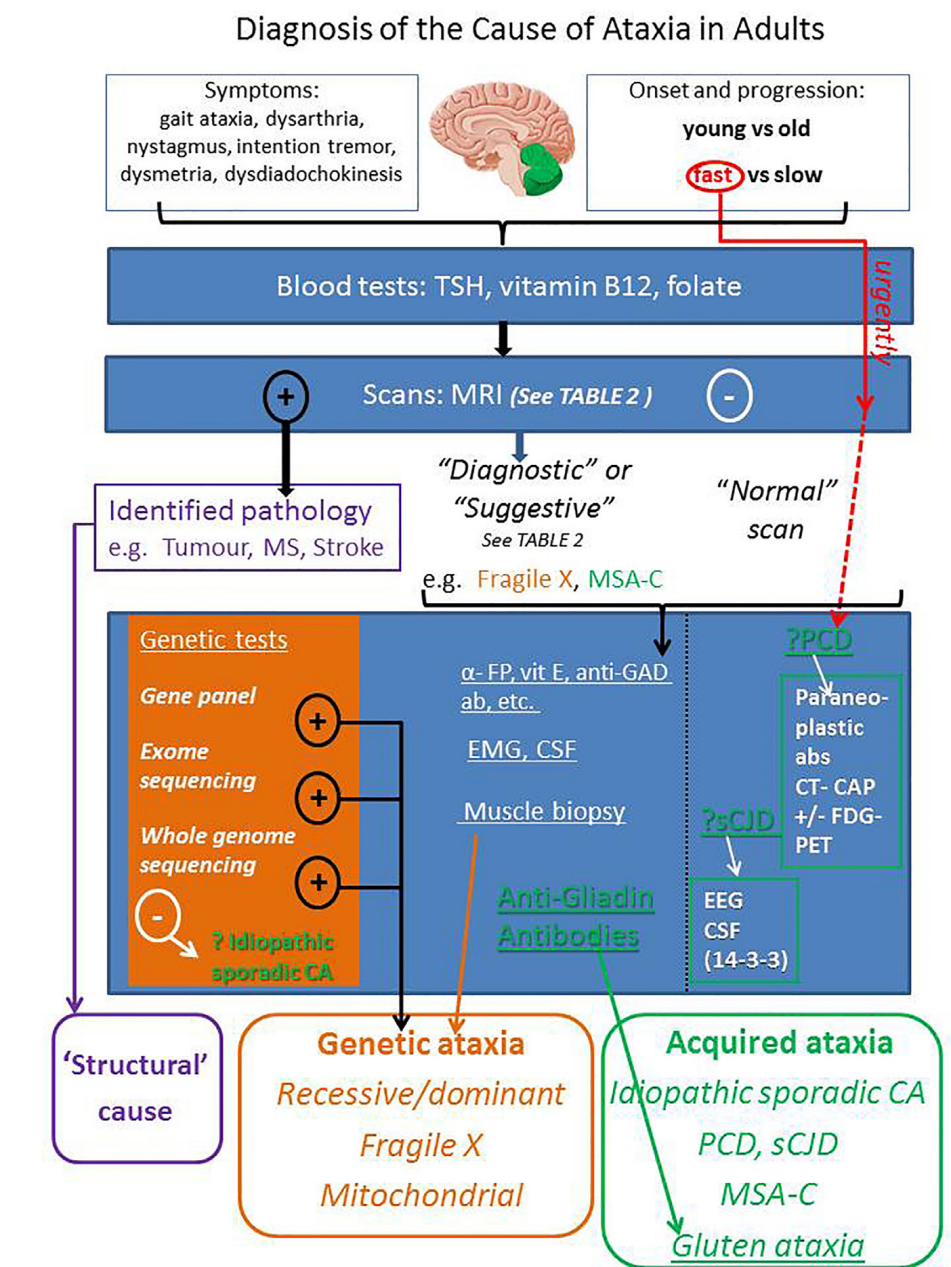
Infographic 1: The Diagnostic Pathway

The diagnostic pathway is summarised in the infographic (figure 8).

### Management

Neurologists traditionally have taken a nihilistic view to managing ataxia, as with many other neurodegeneration diseases. However, this is no longer justified. In addition to rehabilitation therapies, there are specific complications of ataxia to seek and address. These interventions can significantly alleviate the problems of progressive ataxia and prevent complications, which are even potentially life threatening. Finally, there are some rare but treatable conditions that need identifying early. An enthusiastic and well-informed clinician can provide valuable support to a patient with ataxia. Participation in research can be important for patients and, when appropriate, they should be offered opportunities for this at each clinical review (www.clinicaltrials.govand www.ataxia.org.uk). When a patient with ataxia approaches end of life, specialist palliative care services should be involved to help address their specific needs.

### Medical interventions

#### Treatable ataxias

Ataxias that are treatable are rare, except for gluten ataxia and other immune-mediated ataxias. In people with ataxia associated with antigliadin (and possibly more specific antibodies), we recommend a gluten-free diet *even in the absence of enteropathy*.^14^ The antibody titres should be repeated every 6 months to confirm their elimination (by strict adherence to the diet). Note, however, that the symptoms may not stabilise or improve for up to a year.

Ataxia with vitamin E deficiency mimics Friedreich’s ataxia (clinically and on MRI) and can be confirmed genetically. The serum vitamin E concentration is significantly low but should be tested as ‘lipid-adjusted vitamin E’, as free vitamin E concentrations are unreliable and potentially misleading. Malabsorption of vitamin E, including in abetalipoproteinaemia, can result in a similar phenotype. Patients may need replacement doses of up to 1500 mg/day.^15^

Ataxia with CoQ10 (or ubiquinone) deficiency is probably under-recognised. It is a recessively inherited disorder (*ADCK3* mutations) where patients have a low concentration of CoQ10 in skeletal muscle.^16^ Its severity is variable and some people have seizures and mild mental retardation. CoQ10 supplementation can potentially improve the ataxic symptoms, although not in everyone (and the exact form and dose of CoQ10 remains uncertain).^17 18^ Patients with mutations in *APTX* (AOA1) and *ANO10* can develop secondary CoQ10 deficiency, and supplementation may also help them.^19^

Patients with cerebrotendinous xanthomatosis may develop chronic diarrhoea in infancy, and cataracts in the first decade. There may be visible deposits of cholestenol in tendons. If confirmed (usually biochemically), then chenodeoxycholic acid treatment can stabilise or partially reverse the symptoms, or (if given very early) may even help the neurological complications.^20 21^

Niemann-Pick type C is a multisystem disorder caused by the accumulation of cholesterol and glycosphingolipids in the brain and other organs. Consequently, patients may develop splenomegaly and hepatomegaly as well as ataxia. There may be a vertical supranuclear gaze palsy, sometimes with dystonia, myoclonus, epilepsy and cognitive decline. The diagnosis can be challenging, especially in (atypical) cases that present later in life, and biopsy (of bone marrow and skin) does not always give a definitive answer.^22^ Genetic testing for the two causative genes (*NPC1* implicated in 95% and *NPC2* implicated in 5%) may be more reliable. Plasma oxysterols and bile acid measurements may prove to be useful and inexpensive screening tools.^23–25^ Miglustat is an approved disease-modifying therapy for patients with Niemann-Pick type C.^26 27^

There are other treatable causes of ataxia, but these typically present in children and are usually managed in paediatric practice. These include CoQ10 (or ubiquinone) deficiency, hypobetalipoproteinaemia, Hartnup disease, biotinidase deficiency and pyruvate dehydrogenase deficiency. Glucose transporter 1 deficiency often presents with paroxysmal movement disorders, spasticity and ataxia, and its onset can be delayed. A ketogenic diet is effective in treating the associated epilepsy but perhaps is less effective in helping the gait difficulties.^28^

#### Symptomatic treatments

**Figure 9:**
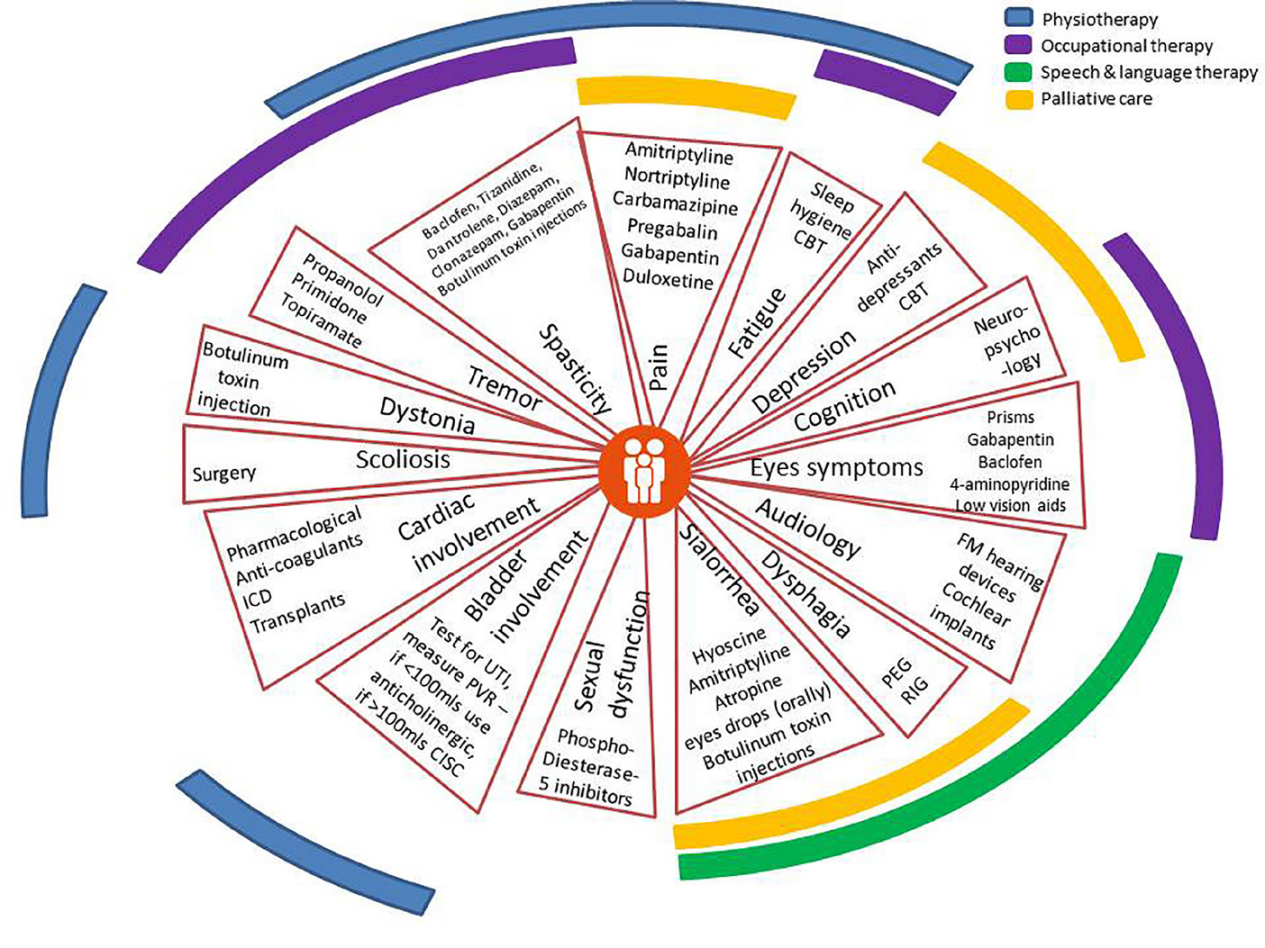
Infographic 2: Symptom Management, including Multidisciplinary Team Input

The infographic summarises the potential symptoms that neurologists may need to address (figure 9). The treatment ‘strategies’ are frequently derived from other neurological conditions with similar symptoms, and generally work equally well. The approach to treating spasticity and bladder symptoms, for example, is the same as for people with multiple sclerosis.^29 30^ The assessment and management of these complications are best done by involving therapy specialists, and multidisciplinary team working can greatly enhance patient care. Speech and language therapy input is essential throughout the patient journey, from monitoring swallowing function in the early stages and providing helpful hints on avoiding complications, to planning percutaneous gastrostomy feeding.^31^ Note that almost all these interventions to manage patients with ataxia are non-evidence based, and will probably never be studied in high-quality randomised controlled trials.

The impact of cerebellar disease on cognition is not widely known but can significantly impact on morbidity. Such ‘remote’ effects of cerebellar dysfunction can include subcortical frontal impairments, affecting personality, behaviour and judgement.^32^ Mental health complications (anxiety, depression) may exacerbate people’s sense of isolation and fear of the future. These symptoms often accompany sleep disorders and fatigue but are poorly recognised (according to Ataxia UK’s survey). The management of cardiac complications is especially important in Friedreich’s ataxia; patients need regular ECGs and (ideally) echocardiograms to detect cardiomyopathy developing.^33–35^ Echocardiography may show concentric left ventricular hypertrophy (possibly in over half of cases, especially in those of early onset). With disease progression, the hypertrophy regresses, resulting in a thin and dilated left ventricle. Serum troponin may be asymptomatically elevated (in the absence of arrhythmia or an acute coronary syndrome), and it may help to have baseline values for future comparison. It is essential to involve an interested and knowledgeable cardiologist with experience of the care of Friedreich’s ataxia, initially to advise on medication to treat the cardiomyopathy and heart failure, and later for the management of arrhythmias.

Episodic ataxia type 2 is characterised by periods of cerebellar dysfunction lasting for hours or days, sometimes with migraine, and rarely epilepsy. Later in life, the ataxia becomes progressive, and MRI may show cerebellar atrophy.^36^ Attacks can be precipitated by stress, exertion, caffeine and alcohol, and patients should be counselled appropriately. Acetazolamide remains the mainstay of treatment but carries a risk of renal calculi and (more commonly) of paraesthesia. As well as good hydration, we recommend annual ultrasound scans of the urinary tract. Flunarizine and 4-aminopyridine, a potassium channel blocker, can also help but (like acetazolamide) they are not licensed in the UK to use in episodic ataxia type 2.^37^ Dichlorphenamide is extremely effective and well tolerated but is currently too expensive to use routinely. Having seizures is a contraindication to using 4-aminopyridine.

### Allied health professional interventions

The multidisciplinary team is clearly important in evaluating and managing patients with ataxia. Speech and language therapy (for both communication and swallowing), occupational therapy and physiotherapy can each make important contributions at different stages of the patient journey. It can be difficult to access interested and knowledgeable therapists in the community, and this type of expertise is rare outside of specialist ataxia clinics. The guidelines have sections in each of these fields, giving comprehensive advice on the types of assessments and interventions that need to be done, and may serve as an educational resource to accompany referrals to community therapy teams. In our experience, the nurturing of local ‘champions’ in ataxia management (frequently a therapy colleague working in a district general hospital) can generate enthusiasm and help develop local expertise; in time these therapists frequently become effective access points for services for patients and their carers.

#### Reviews and monitoring

Patients with progressive ataxias should be offered 6–12 monthly reviews, ideally by a general neurologist or (where available) an ataxia specialist (neurologist or nurse). There are also services specifically for adults and children with ataxia-telangiectasia, where specialist input (again by multiprofessional teams) can tackle the complications associated with this condition, including cancer predisposition, immunodeficiency and lung disease. Patients require regular review to identify any new symptoms early that may need treatment, and for patients to take advantage of advances in diagnostics and any new available treatments. In addition, those with no established cause for their ataxia can undergo thorough and repeated review of the clinical features and investigation results, which sometimes leads to a clearer diagnosis.

#### Patient support groups

Patients and their families should be encouraged to contact patient support groups, such as Ataxia UK. When a family first receives the diagnosis of progressive ataxia, patients are usually not heard of the condition or come across other people with it. Support from patient organisations can therefore be particularly important at this stage. The possibility of meeting others in the same situation, receiving emotional support and information, and the opportunity to learn of research developments (as well as taking part in research projects) can all help.

#### Palliative care

Given that most progressive ataxias are incurable, there are strikingly few published studies on their palliation and end-of-life care. Many patients with progressive ataxia have a normal life expectancy but some forms (eg, multiple systems atrophy type C) can progress rapidly, with a shortened life. The recommendations in these guidelines are drawn from the wider field of progressive neurological conditions.

We suggest that patients discuss advanced care planning at the appropriate time. Patients with intractable and/or distressing physical symptoms may benefit from referral for specialist palliative care, which might also help those with complex social, psychological or spiritual needs. The time for planning end-of-life care is when the clinician answers ‘No’ to the ‘surprise question’—‘Would you be surprised if this patient died in the next 12 months?’—as well there being generic and specific (for ataxia) indicators that the patients have reached the terminal phase of their illness. Management in this phase should be geared towards enabling a ‘good death’: being treated as an individual, with dignity and respect, without pain or other distressing symptoms, in familiar surroundings, and in the company of close friends and family.^38^

**Key points**

- Progressive ataxia in adults is heterogeneous and can be difficult to diagnose.
- Brain imaging, ideally with MRI, is essential.
- Rapidly progressive ataxia warrants urgent investigation (particularly to exclude underlying malignancy).
- Next-generation sequencing is enabling many more ‘idiopathic’ ataxias to be given a genetic cause.
- Immunity may be an under-recognised (and potentially reversible) cause of progressive ataxia.
- The complications accompanying certain ataxias (eg, Friedreich’s ataxia with cardiomyopathy and diabetes) require active monitoring and management.
